# How far should I manage acute optic neuritis as an ophthalmologist? A United Kingdom perspective

**DOI:** 10.1038/s41433-024-03164-4

**Published:** 2024-06-12

**Authors:** Heidi Laviers, Axel Petzold, Tasanee Braithwaite

**Affiliations:** 1 The Medical Eye Unit, Guy’s and St Thomas’ NHS Foundation Trust, London, UK; 2 Department of Ophthalmology, King’s College Hospital, London, UK; 3 Neuro-ophthalmology Service, Moorfields Eye Hospital, London, UK; 4 Neuro-ophthalmology Service, The National Hospital for Neurology and Neurosurgery, London, UK; 5 Amsterdam University Medical Center (AUMC), Amsterdam, Netherlands; 6 The School of Immunology and Microbial Science and The School of Life Course and Population Sciences, King’s College London, London, UK

**Keywords:** Optic nerve diseases, Central nervous system infections, Autoimmune diseases, Inflammatory diseases, Eye manifestations

## Abstract

Optic neuritis (ON) is an inflammation of or around the optic nerve, frequently caused by infectious or immune-mediated inflammatory disorders. In the UK, its strongest association is with Multiple Sclerosis (MS), though the combined prevalence of other associated infectious and immune-mediated inflammatory diseases (I-IMID) is similar to that of MS-ON. Prompt identification and understanding of ON’s underlying cause informs tailored management and prognosis. Several IMIDs linked to ON, such as aquaporin-4 antibody-associated optic neuritis (AQP4-ON), myelin oligodendrocyte glycoprotein antibody-associated optic neuritis (MOG-ON), and neuro-sarcoidosis, show remarkable response to corticosteroid treatment. Therefore, urgent investigation and treatment are crucial in cases ‘atypical’ for MS-ON. Following the 1992 Optic Neuritis Treatment Trial, clinical practice has evolved, with short-course high-dose corticosteroids considered safe and effective for most people. Timely recognition of patients who could benefit is critical to avoid irreversible vision loss. This review provides a practical guide and a summary of evidence on the investigation and management of acute optic neuritis. It reflects the knowledge and limitations of current evidence, framed through the neuro-ophthalmic perspective of clinical practice at multiple UK academic centres.

## How do I spot an acute optic neuritis?

The presence or absence of symptoms in optic neuritis (ON) depends on the site of inflammation within the anterior visual pathway, and how long after symptom onset a patient presents. The optic nerve does not contain pain receptors. However, patients with retrobulbar optic neuritis (ON) may experience pain with eye movement on account of traction on the optic nerve sheath stimulating nociceptive afferents of trigeminal origin where the sheath adheres to the superior and medial recti at the orbital apex [1]. Pain often precedes vision change, and is characteristic of Multiple Sclerosis (MS)-associated ON, and ON associated with anti-myelin oligodendrocyte glycoprotein antibodies (MOG-ON) and anti-aquaportin-4 antibodies (AQP4-ON). In other ON subtypes, vision impairment is more often painless on account of more anteriorly localised inflammation proximal to the lamina cribrosa, or inflammation of the intracranial portion of the optic nerve after it has parted with the optic nerve sheath. A prodromal viral illness may be reported. Patients may report changes in colour vision, with reds appearing ‘washed out’ or ‘faded’. Some patients may note positive symptoms of photopsia. The neurological functional enquiry or medical history may offer clues, such as symptoms of transient, temperature-dependent worsening of visual function (Uhthoff phenomenon) or difficulties with perception of movement in depth (Pulfrich phenomenon). In MS-ON, transient blurry or double vision on horizontal eye movement, may be indicative of internuclear ophthalmoplegia. Prior episodes of numbness, weakness, cognitive dysfunction, and bladder or bowel dysfunction, lasting more than 24 h and resolving within weeks, are also suggestive of MS. More sinister neurological symptoms might include transverse myelitis, or hiccups (area postrema syndrome in aquaporin-4 antibody-associated (AQP4) disease), or symptoms of a brainstem lesion. Fortunately, such patients are more likely to present to the main emergency department. Classically, acute vision loss in MS-ON worsens over days, reaching a nadir around two weeks, gradually improving back towards baseline high contrast visual acuity by three months [2, 3]. All the other causes of ON (which we will abbreviate here to ‘Non-MS-ON’) may mimic MS-ON, or present with more significant vision loss at onset, even to bilateral ‘no perception of light’.

It is important for ophthalmologists to appreciate that thorough investigation of ON in the weeks following first eye casualty presentation permits more nuanced ON subclassification at the conclusion of neuro-ophthalmic assessment. International consensus currently recommends the following ON subcategories: MS-ON, MOG-ON, AQP4-ON, isolated ON (ION), relapsing ON (RION), and chronic relapsing inflammatory ON (CRION) [3]. In addition, ON may develop as part of anti-glial fibrillary acidic protein (GFAP) antibody-associated meningoencephalitis, and in association with anti-glycine receptor a1 subunit antibodies [4].

## Clinical signs of optic neuritis

The first episode of unilateral ON results in a relative afferent pupil defect, red desaturation and blurred vision, with or without a visual field defect. Frequently, a central scotoma is noted, but depending on the site of inflammation, any visual field defect can be observed [3]. The disc may be swollen in one third of MS-ON, and a higher proportion of non-MS-ON [3, 5–7]. Non-MS-ON has more heterogeneous presentation, this is particularly true for MOG-ON [4, 5, 8, 9].

## Optical coherence tomography (OCT) imaging findings in optic neuritis

OCT imaging of the peripapillary retinal nerve fibre layer (pRNFL) aids identification of subtle optic disc swelling. OCT segmentation at baseline and follow-up valuably detects an inter-eye difference in the macular ganglion cell inner plexiform layer (mGCIPL) of > 4% or > 4μm, or in the pRNFL of > 5% or > 5μm, within 3 months of onset [3]. Statistically, the inter-eye percentage difference (%) outperforms the absolute difference(μm). In the acute setting, thinning of the pRNFL or mGCIPL indicates either a longer duration, or a prior episode, because these changes take at least a month to evolve. Quicker rates of atrophy are more typical for an ischaemic optic neuropathy [10].

## How likely am I to see acute optic neuritis in a UK eye casualty?

Using primary care record data from 11 million NHS patients with 75 million person-years of follow-up (1997–2018), we previously estimated a stable UK-ON incidence of 3.7 (95% confidence interval 3.6–3.9) per 100,000 person-years [8]. The incidence in children aged 1 to 17 years was 0.8 (95% CI 0.6–0.9), and in adults was 4.5 (95% CI 4.3–4.6), per 100,000 person-years [8]. This equates to an estimated 2894 new ON cases presenting to eye casualties across the UK each year. In 2018, there were an estimated 76,279 patients who had ever had ON (prevalence 115 per 100,000).

## Which patients are most at risk of ON?

Understanding the demographic and other clinical characteristics typical for ON informs the prior probability of the diagnosis being ON, rather than other possibilities in the differential, and guides appropriate investigation and management of potentially modifiable risk factors. We have previously reported that Scotland residents were 1.2-fold more likely than England residents to develop incident ON (Incidence rate ratio (IRR) 1.22, *p* < 0.001), likely relating to the known effect of latitude on MS incidence [8]. Women were 2.3-fold more likely to develop ON (IRR 2.26, *p* < 0.001). People of South Asian (IRR 0.56, *p* = 0.001) or mixed race (IRR 0.52, *p* = 0.02) were half as likely as white people to develop ON. People living with obesity (IRR 1.27, *p* = 0.001) or morbid obesity (*p* < 0.001) were at heightened ON risk compared to those with normal weight. Current smokers (IRR 1.32, *p* < 0.001) and ex-smokers (IRR1.19, *p* = 0.007) were also at elevated risk compared to never smokers. Whilst young women in their reproductive years, and those born at higher latitude were more likely to present with MS-ON, non-MS-ON can present in any age, geographic location, or race [8].

## How likely is it that ON relates to MS?

At first ever ON presentation in the UK, 67% of cases are undifferentiated, whilst 33% have history of a prior infectious or immune-mediated inflammatory disease (I-IMID) [8]. Importantly, 15.5% have a prior I-IMID diagnosis, 14.3% have a prior MS diagnosis, and 3.0% have both [8]. Past history of any given I-IMID is rare, but collectively, these non-MS associations are as frequent as prior MS history.

Amongst the initially undifferentiated ON cases, 22.4% eventually develop MS, compared to 0.1% controls (adjusted Hazard Ratio (aHR) 284.97, *p* < 0.001). Differentiation from those who may have an underlying corticosteroid-responsive cause for their ON is critical to effective sight-saving management in the latter group. It is particularly important to identify patients whose initially undifferentiated ON results from immune suppression-requiring AQP4-disease (1.2% cases), sarcoidosis (0.5% cases versus 0.03% controls, aHR 14.8, *p* < 0.001), Behcet’s disease (0.1% cases versus 0.01% controls, aHR 17.39, *p* = 0.02), and other vasculitides (besides giant cell arteritis)(0.3% cases versus 0.05% controls, aHR 4.89, *p* = 0.002) [8]. Undiagnosed infectious causes of undifferentiated ON are much less common than autoimmune and autoinflammatory causes of ON in the UK, with herpetic infection being the most common, in 1.8% cases versus 1.1% controls (aHR 1.68, *p* < 0.001) [8]. ON may also be associated with certain drugs including the anti-TNFa biologics (e.g. etanercept), immune check-point inhibitors (eg Ipilimumab) and may appear to follow vaccination [3, 11], though whether these are truly causal is uncertain.

## The differential diagnosis of optic neuritis

Diagnostic error is common in patients with suspected ON, and can cause patient harm [12, 13]. One neuro-ophthalmology centre in the USA reported that 60% (73/122) of patients referred with suspected acute ON did not have ON [12]. The most common alternative diagnoses were primary headache disorder with associated eye pain (22%), functional vision loss (19%), other optic neuropathies (16%), particularly non-arteritic anterior ischaemic optic neuropathy (NAION), and other conditions including neuroretinitis, retinal artery occlusion, and optic nerve sheath meningioma. Amongst patients with MS, 4% had an alternative diagnosis to ON. A careful history and clinical examination, considering the time course of pain and vision loss, and weighing and interpreting clues, advances the differential, and helps target investigations and treatment appropriately. Table 1 illustrates a non-exhaustive differential diagnosis. Stunkel et al. highlighted that normal examination findings are reassuring and virtually exclude a diagnosis of ON, especially the absence of a relative afferent pupil defect in suspected unilateral ON [12].

**Table 1:** Conditions to consider in the differential diagnosis for optic neuritis, informed by the presence or absence of pain on eye movement, disc swelling and a relative afferent pupil defect.

	Disc swelling	No disc swelling
**Painful**	Posterior/pan/intermediate uveitis	Posterior/pan/intermediate uveitis Scleritis Orbital myositis Orbital inflammation Primary headache disorder Functional vision loss
**Painless**	Non-arteritic anterior ischaemic optic neuropathy^a^ Arteritic anterior ischaemic optic neuropathy (+/- headache)^a^ Neuroretinitis Leber’s hereditary optic neuropathy^a^ Malignant hypertension Diabetic papillitis Posterior/pan/intermediate uveitis	Posterior ischaemic optic neuropathy secondary to vasculitis, prone surgery, or uncontrolled hypertension^a^ Retinal artery occlusion^a^ Other retinal pathologies which might be overlooked: acute macular neuroretinopathy, paracentral acute middle maculopathy, acute zonal occult outer retinopathy, acute idiopathic blind spot enlargement syndrome Optic nerve sheath meningioma^a^ or posterior ethmoid mucocele causing compressive optic neuropathy^a^ Elevated intracranial pressure (± headache) Crowded optic discs (pseudo-swelling) Optic disc drusen (pseudo-swelling) Functional vision loss Posterior/pan/intermediate uveitis

^a^RAPD expected if unilateral symptoms.

This table is provided for illustration and is not exhaustive. Less typical presentations may not follow this simplified categorisation.

There are numerous causes of unilateral or bilateral optic disc swelling. In anterior ischaemic optic neuropathy (AION), onset is usually abrupt, painless and unilateral. Non-arteritic AION most typically presents in patients over 50 years of age, with altitudinal hemifield loss first noticed upon waking, and the unaffected side has a ‘disc at risk,’ crowded configuration. In arteritic AION, patients may have symptoms of giant cell arteritis, reduced temporal artery pulses, and elevated CRP, ESR and/or platelets. Relatively swift, painless vision loss in a young man, with asymmetric but bilateral disc swelling (then atrophy) may be Leber’s hereditary optic neuropathy. In uveitis, thickening of the retinal nerve fibre layer may be noted on OCT imaging, without relative afferent pupil defect. Diabetic patients with poor glycaemic control develop asymptomatic ‘diabetic papillopathy’, usually with good visual function. Malignant hypertension usually causes bilateral disc swelling and subsequent partial optic atrophy, sometimes without other signs of hypertensive retinopathy. Elevated intracranial pressure, typically associated with bilateral disc swelling, often presents with an enlarged blind spot and nasal-step on visual perimetry but typically lacks red desaturation or central field loss. There are many causes of elevated intracranial pressure, but those with abrupt onset, such as cerebral venous sinus thrombosis, may be more likely to be misdiagnosed as bilateral ON. Errors in pRNFL scan centring or segmentation can mistakenly suggest swelling, as acknowledged in validated quality control criteria (OSCAR-IB) [14]. Apparent swelling and elevation of the optic disc without significant pRNFL thickening can be observed in cases of peripapillary hyperreflective ovoid mass-like structure (PHOMS), in patients with chronic raised intracranial pressure, optic disc drusen, or anatomical optic disc crowding [15].

Similarly, painless optic nerve pathologies without disc swelling may be mistaken for acute ON. The clinical history will identify patients at risk of a traumatic or drug-induced or metabolic syndrome optic neuropathy, or post-surgical posterior ischaemic optic neuropathy (PION). This is seen rarely in patients following protracted surgery, most commonly associated with coronary artery bypass grafting and back or spinal surgery [16–18]. The presumed cause is ischaemic hypoperfusion, and a number of risk factors have been reported in the literature including anaemia, intraoperative blood loss, operative duration, and low blood pressure. Symptoms are typically reported within 1–2 days of surgery and frequently upon awakening, although this may be delayed in those sedated after surgery. Presentation is usually with painless central or peripheral vision loss or both, decreased or absent colour vision which is bilateral in over 60% of cases [16]. Rarely, PION presents in patients with occult giant cell arteritis (GCA), malignant hypertension, or other vasculitides. In GCA, PION occurs in less than 3–6% of cases and presents as sequential vision loss [19]. The optic disc examination is normal in the acute setting, with the development of optic atrophy approximately 6 weeks after the event [19]. Functional enquiry may reveal other symptoms suggestive of vasculitis, and inflammatory markers and anti-neutrophil cytoplasmic antigen (ANCA) may be abnormal. Patients sometimes suddenly notice the monocular vision impairment associated with a compressive optic nerve lesion such as a mucocele, or optic nerve sheath meningioma, which may be mistaken for ON prior to neuroimaging [20]. Scleritis, myositis and orbital inflammation may result in pain on eye movement which could mimic ON without optic disc swelling, but in these conditions, there are usually other clinical signs, and no colour desaturation or RAPD (unless there is compression or concurrent ON).

Cone dysfunction will also impair colour perception and other subtle retinal pathologies may impair visual acuity (e.g. acute macular neuroretinopathy, paracentral acute middle maculopathy) or visual field (e.g. acute zonal occult outer retinopathy, acute idiopathic blind spot enlargement syndrome). Multimodal imaging (with optical coherence tomography, autofluorescence, and even fundus fluorescein and indocyanine green angiography) valuably aids identification of alternate pathologies. Electrodiagnostic testing with 30 Hz flicker, multifocal ERG and visual evoked potentials can also helpfully differentiate primary retinal from optic nerve pathology, but may not be available in the acute setting.

### Management

#### The first decision the ophthalmologist needs to make when ON is suspected: Does this patient need to be admitted today?

Patients with acute or recent onset bilateral moderate to severe vision impairment require consideration of acute medical admission. This can also be considered for patients with severe monocular vision loss, or other factors precluding safe out-patient management. Patients with low vision and sluggish or unreactive pupils are of particular concern for possible AQP4-ON, MOG-ON, or severe post-viral ON. Focal neurology may advance the differential diagnosis. These patients will require urgent investigation including neuroimaging (see Table 2), and intravenous methylprednisolone (IVMP) 1 g once daily for 3 to 5 days. Given the time needed to arrange medical admission, we usually offer a STAT high dose of oral methylprednisolone, but note this might mask subtle MRI nerve enhancement. The patient will typically have early review by the on-call neurology team, and/or neuro-ophthalmologist for consideration of lumbar puncture (LP). If there is poor initial response to IVMP, Plasma Exchange (PLEX) may be considered. This is particularly effective in AQP4-ON and MOG-ON [3, 21].

**Table 2:** Our typical approach to investigation of optic neuritis in the United Kingdom.

Investigation	Rationale	Core or extended
Imaging		
Computerised Tomography Head with contrast (+/- orbits)	For rapid exclusion of a space occupying lesion prior to initiation of corticosteroid therapy (e.g. posterior ethmoid mucocele), especially in patients with history or symptoms of sinus-related disease. Adding an orbit imaging protocol offers more useful information but is generally not available as a same-day imaging investigation in the UK.	Extended
MRI brain and orbits with orbital fat suppression and gadolinium contrast	T2 hyperintensity and gadolinium-enhancement on fat-suppressed MRI images of the brain and orbits are sensitive for the detection of optic neuritis in most cases. Generally available within 24–48 h for inpatients and within 2 days to 2 weeks for urgent outpatients	Core
Chest radiograph	To screen for sarcoidosis (0.9% UK ON cases) and pulmonary tuberculosis. Required prior to initiating corticosteroid-sparing immunosuppression.	Core
Blood tests		
Full blood count	To screen for systemic infection. Elevated platelet count may be seen in giant cell arteritis. Reduced lymphocyte count may be seen in sarcoidosis. Required prior to initiating corticosteroid-sparing immunosuppression. Significant anaemia may be associated with pseudotumour cerebri (differential diagnosis for ON)	Core
Renal profile	Required prior to administration of gadolinium, on account of the very rare but increased risk of Stiff Person Syndrome if the eGFR is significantly reduced below 30-40 ml/min/1.73m^2^. Elevated creatinine/reduced eGFR may be seen in patients with renal vasculitis or renal impairment associated with diabetes, hypertension, sarcoidosis, vasculitides and other conditions.	Core
Liver function tests	Elevated alkaline phosphatase may be seen in sarcoidosis (0.9% UK ON cases). Required prior to initiating corticosteroid-sparing immunosuppression.	Core
Bone profile	Elevated corrected calcium may be seen in sarcoidosis (0.9% UK ON cases). Calcium supplements are avoided in these patients. Hyper- or hypo- calcaemia may be associated with pseudotumour cerebri (differential diagnosis for ON).	Core
Serum angiotensin converting enzyme	May be elevated in sarcoidosis (0.9% UK ON cases).	Core
C reactive protein	Non-specific, but may be elevated in many immune-mediated inflammatory disorders and systemic infections. For example, CRP may be elevated in Crohn’s disease (0.9% UK ON cases), sarcoidosis (0.9% UK ON cases), and vasculitides.	Core
Erythrocyte sedimentation rate	Non-specific, but may be elevated in vasculitides. 1.5% UK ON cases are associated with giant cell arteritis (GCA) and a further 1% with other vasculitides.	Core
Anti-neutrophil cytoplasmic antibody	Non-GCA vasculitis, including granulomatosis with polyangiitis, accounts for approximately 1% of UK ON cases	Core
Treponema serology ± Rapid plasma reagin	Positive treponema serology indicates previous exposure to treponema species; a positive RPR indicates active syphilis infection. Syphilis is associated with 0.2% UK ON cases (higher risk in certain population subgroups).	Core
Human immunodeficiency virus	HIV is an extremely rare cause of optic neuritis, but we routinely test this in the emergency department to avoid overlooking atypical presentations involving opportunistic infection.	Core
Vitamin B12, serum folate and homocysteine	We routinely check these for the purpose of secondary prevention of further progressive optic neuropathy, and this is especially important in patients with high alcohol intake or conditions associated with gastrointestinal malabsorption or dietary malnourishment.	Core
Anti-aquaporin 4 antibody and anti-myelin oligodendrocyte glycoprotein antibody	Neuromyelitis optica accounts for approximately 1.4% cases of UK ON cases. Requires neuroimmunology lab ‘send away’ of a serum sample	Core
Vitamin D	Vitamin D deficiency is a risk factor for multiple sclerosis, associated with at least 36% ON in UK	Core
Thiopurine methyltransferase (TPMT) level	Required prior to initiating corticosteroid-sparing immunosuppression with azathioprine	Extended
Epstein Barr virus IgG	3.8% UK ON cases have prior history of glandular fever or EBV infection, an established causal risk factor for MS-ON	Extended
Hepatitis C IgG, Hepatitis B core antibody, Hepatitis B surface antigen	Required prior to initiating corticosteroid-sparing immunosuppression	Extended
Anti-nuclear antigen and Extractable Nuclear Antigen Panel	In UK, 0.3% ON associated with Sjogren’s syndrome with rarer association to some other rheumatological conditions	Extended
Herpes simplex virus and varicella zoster virus IgG and IgM	5.8% of UK-ON cases have preceding herpetic infection. Seropositivity is high in the general population, so these are most helpful if negative in selected cases	Extended

#### If the patient does not need to be admitted today, how far should I go to investigate acute optic neuritis as an ophthalmologist?

The positive and negative predictive value of a test are influenced by the prevalence of the disease in the population being tested. Our practice is informed by epidemiological insights into significant ON-disease associations in the UK population (see Table 2) [8]. After consulting a neuro-ophthalmology Consultant, our eye casualty team may request additional specialised tests, in line with the International Classification of Optic neuritis (ICON) 2022 diagnostic criteria [3]. Serum MOG-IgG and AQP4-IgG assays appear to be more sensitive than CSF assays [22, 23]. The urgency of the MRI depends on the suspected condition: urgent for non-MS-ON cases and routine for MS-ON. This early discussion helps minimise unnecessary requests for ‘urgent’ neuro-ophthalmology out-patient review, and optimises the use of limited service capacity. Our goal is to see out-patients within a week of starting corticosteroid treatment to assess response.

We sometimes consider outpatient LP, guided by the neuroradiology findings. Cerebrospinal fluid (CSF)-restricted oligoclonal IgG bands (type 2 pattern), indicating intrathecal IgG synthesis, are a paraclinical test for MS [24]. Differentiation between MS and neuro-sarcoidosis can occasionally be difficult. CSF analysis also helps us investigate rarer infective, neoplastic and paraneoplastic causes. Where the latter is suspected, additional serum tests (e.g. for collapsin response mediator protein-5) and CT positron emission tomography imaging are considered.

#### Time is Vision: The case for early initiation of treatment by an ophthalmologist in ON patients not requiring acute medical admission

In our centres, knowing that an urgent MRI will be performed soon (within 2 days to 2 weeks), we generally do not await the MRI result to start high-dose corticosteroid therapy (see next section) in suspected or relapsed non-MS-ON cases, in cases of severe vision loss, or second eyes. There are three key reasons for this. Firstly, we take the perspective that ‘time is vision’, and that a short course of high-dose corticosteroid in patients with no contraindications is generally well-enough tolerated. The relationship between visual acuity and the number of optic nerve axons is non-linear [25, 26]. A healthy nerve contains approximately 1.2–1.5 million axons, and a proportion of these can be lost whilst maintaining 6/6 visual acuity and full colour vision. However, with progressive axonal loss, or significant loss during a single episode, visual function decline will be noticed, especially for tasks involving low contrast, such as reading small print in dim lighting. OCT reveals that after a first episode of MS-ON, the mGCIPL thins by an average of 9.29 ± 7.2 μm (12.2%) at 6 months [27]. It takes 1–3 months for these changes to become apparent, as axonal degeneration propagates back towards the cell body within the macula. Whilst a first episode may not permanently impact visual acuity, with further episodes, cumulative injury may result in a permanent decline once some critical threshold of axons is crossed. Loss of vision in patients with recurrent ON can be prevented if treatment is initiated in the early inflammatory phase, which patients recognise from previous attacks [9, 28, 29]. Studies confirm that inflammation precedes demyelination and axonal degeneration by approximately 2 days. Irreversible damage to the axonal cytoskeleton occurs within 5–7 days [9]. The critical time interval for treatment initiation is therefore within 48 h [9, 28–30].

Secondly, it is important to note that while MRI imaging generally reveals demyelination effectively, older studies reported that 60% of ON patients had normal MRI [3, 31]. This contrasts with later literature, which predominantly focuses on MS-ON and adheres to established radiological criteria for MRI acquisition and interpretation [3, 32, 33]. In everyday clinical practice, the effectiveness of MRI depends on the location of the optic nerve involvement and the imaging protocol and quality [2]. MRI is highly effective for imaging stationary parts of the optic nerve up to the chiasm, but cannot detect pre-laminar ON (inflammation anterior to the lamina cribrosa) [3].

Thirdly, in the UK population, the risk of treating an undifferentiated ON which is driven by an infectious disease process is low. Typically, serology results for infectious diseases come back in time to prevent potential long-term complications from unopposed corticosteroid treatment.

#### Corticosteroid treatment for suspected or confirmed MS-ON?

We also take the ‘Time is Vision’ perspective in suspected MS-ON. The 1992 landmark Optic Neuritis Treatment Trial (ONTT) historically guided the treatment of acute monocular ON in a certain patient demographic [2, 6, 7]. The trial recruited 457 patients, 77% female, 85% white, with a mean age of 32 years. There have been calls for a new ONTT to address limitations and evidence gaps [9]. Re-analysis revealed inclusion of a small number of patients with MOG-ON, a corticosteroid-sensitive and sometimes even corticosteroid-dependent ON [3, 9]. Few recruited patients were treated in the hyperacute phase ( < 48 h from pain onset) with a mean time to treatment of 5$\pm$1.6 days after visual loss, and so the benefits of early corticosteroid treatment were not assessed. The ONTT also used high contrast visual acuity as the primary outcome measure. More sensitive visual function tests include Low Contrast Visual Acuity (LC-VA) and Colour Assessment and Diagnosis (CAD). Furthermore, the ONTT recruited a heterogeneous ON cohort, but findings were not generalisable to all ON patients, and especially to patients with more severe or bilateral non-MS-ON.

We ask our eye casualty doctors to counsel patients about the evidence from the ONTT, and the side effects and contraindications to corticosteroid therapy (Box 1), in order to offer patient choice around starting a short course of methylprednisolone, especially when the patient presents within 48 h of symptom onset. We typically co-prescribe a calcium and vitamin D supplement for bone protection, and a proton pump inhibitor for gastric protection. We ‘safety net’ the patient to return to eye casualty if their vision continues to decline after 2 weeks or if they have any new concerns.

According to the ICON 2022 consensus paper, experts acknowledged a lack of consensus on the optimal dosage of corticosteroids, with no randomised controlled trial evidence favouring one dosage over another [3]. Local experience typically dictates the chosen dosage, with some centres preferring 1.25 g methylprednisolone orally for 3 days, and others 500 mg orally for 5 days. There is evidence that IVMP is better tolerated than oral, particularly with regard to insomnia, but arranging the nursing staff and infusion bed can be challenging [34, 35]. This was particularly true during the COVID-19 pandemic, when our practice shifted towards oral therapy for most patients, and this more practically achievable approach has persisted.

Whether you and your teams feel in a position to initiate high dose corticosteroid therapy will depend on local policies, arrangements of emergency eye, neuro-ophthalmology and neurology services.

#### Exceptions: when we do not initiate early corticosteroid treatment

We take a more cautious approach in patients who are immunosuppressed, have poorly controlled diabetes or hypertension, past gastrointestinal issues (particularly ulcers), mental health disorders, and in the elderly or otherwise frail. These patients have a greater risk of experiencing the adverse effects or complications of corticosteroid therapy. In people with MS who are on highly effective immunomodulatory treatment, such as natalizumab, ON is almost never seen, so we carefully seek to exclude alternative ON causes in these patients.

An invasive bacterial or fungal infection causing a compressive optic neuropathy (e.g. mucormycosis) is a rare possibility (Online Supplement Fig. 1). We are guided by additional clinical signs, such as headache, low-grade fever, facial swelling, sinusitis, proptosis, conjunctival injection, ptosis and/ or restrictive ophthalmoplegia [36, 37]. If this diagnosis is suspected, we arrange urgent CT head and orbit imaging with contrast and consider medical admission for systemic antimicrobial therapy and surgical review before taking a decision about corticosteroid treatment.

With regard to other causes of infective ON, we see syphilis most frequently in the MSM (Men who have sex with men) community, and routinely ask about sexual history. In suspected syphilis-ON, the antimicrobial can be started once lab results are back (approximately 3 days), and in our experience, early initiation of corticosteroids followed by the neurosyphilis antibiotic treatment protocol has good visual outcome (Online supplement Fig. 2) [36, 38]. Of note, the National Institute for Health and Care Excellence (NICE) guidelines for the management of neurosyphilis recommend corticosteroid pre-treatment to reduce the risk of a Jarisch-Herxheimer reaction [39].

Herpetic-ON may present concurrent with Herpes Zoster Ophthalmicus (HZO) or develop as a post-herpetic complication later [36]. Adjunct oral corticosteroid with antiviral therapy remains controversial, and the efficacy for herpetic-ON remains unclear, so this is considered on a case-by-case basis [40–44].

Tuberculous(TB)-ON should be considered in the differential diagnosis of apparently isolated papillitis or neuroretinitis in patients from endemic areas, prior TB or TB contact, recent overseas travel ( < 5 years to a high-incidence ( ≥ 100 per 100,000) country), or who have other risk factors for TB reactivation (e.g., HIV infection, diabetes, silicosis, immunosuppressive therapy) [45]. The few studies that have reported on the unopposed use of chronic corticosteroids without anti-TB treatment have reported worse visual outcomes, but corticosteroid therapy may precede initiation of anti-tuberculous therapy in ocular TB, and be used safely as an adjunctive therapy [45–47].

Toxoplasmosis typically presents with hypertensive panuveitis with significant vitreous haze and active chorioretinitis adjacent to an old (hyperpigmented) chorioretinal scar. Reactive optic disc hyperaemia is common. Other clinical presentations include neuroretinitis and isolated anterior optic neuritis [36]. We always avoid unopposed corticosteroid therapy in patients with signs of previous toxoplasma chorioretinitis. Destructive and disseminated ocular toxoplasmosis has been repeatedly demonstrated in case reports after unopposed corticosteroids [48, 49].

We find fundus fluorescein angiography and indocyanine green angiography helpful in unusual ON presentations.

#### How do neuro-ophthalmologists and neurologists manage ON?

Earlier referral to neuro-ophthalmology has been reported, by a 3-centre study in the USA, to have the potential to reduce patient harms associated with misdiagnosis, inappropriate investigation and inappropriate management [13]. However, Neuro-ophthalmologists widely concur (98% agreement) on their diversity of opinions regarding the management of ON! [3] The ICON 2022 diagnostic criteria target readers with a subspecialty interest and suggest more specialised treatment options typically available in expert centers [3]. Monitoring the treatment response to corticosteroids is an essential part of managing ON [3]. In chronic relapsing inflammatory ON (CRION) and AQP4-ON, MOG-ON or ON associated with other IMIDs, following a good response to high-dose methylprednisolone, we consider a subsequent tapering regimen of oral prednisolone. There is no consensus on how long this oral taper should last and we are guided clinically by identifying subtle signs of relapses occurring at dose reduction. For patients who relapse, corticosteroid-sparing immunosuppression is required. Immunosuppressive strategies frequently used to treat AQP4-ON, MOG-ON, neuro-sarcoidosis, and CRION include azathioprine, methotrexate, mycophenolate mofetil, rituximab, and infliximab; rescue immunomodulatory therapies - immunoglobulin and plasma exchange - may also be needed [3]. There is no consensus on when to initiate rescue treatments, and it is necessary to navigate National Health System England commissioned pathways to fund treatment in the UK NHS. We refer all patients with confirmed MS-ON to the MS neurology service for consideration of disease-modifying therapy to reduce the risk of future relapse. Whether these agents have a role to play in clinically isolated ON has not yet been determined [3].

#### Could we enhance risk stratification relating to associated systemic or neurological disease?

Epidemiological evidence has causally linked Epstein Barr virus seropositivity to the onset of MS [50]. Vitamin D deficiency is an additional risk factor [51]. There is also strong genetic predisposition for MS, and for numerous other IMIDs [52, 53]. For example, in MS, genome wide association studies have identified 307 Single Nucleotide Polymorphisms (SNPs) (to date) and strong HLA association [53–55]. We have shown that combining a patient’s sex, age at ON onset, and polygenic risk score for MS into a combined model differentiates UK Biobank participants with undifferentiated ON into a low risk group (4% develop MS) versus a high risk group (44% develop MS), and this model replicates well in independent populations in Finland (FinnGen) and the USA (Geisinger) [53]. We anticipate that in the near future, genetic information will enhance the investigation of patients with ON in the acute setting.

Box 1 Counselling patients around potential side effects of short course high dose corticosteroids for the management of acute optic neuritis We advise patients that corticosteroids can have significant side effects. These include poor sleep, mood change, feeling hungry, gaining weight, reduced sex drive, bone thinning, sudden loss of blood supply to the hip joint, easy bruising, and increased blood pressure and blood sugar levels. The National Institute for Health and Clinical Excellence (NICE) advise that corticosteroids are not a safe option for people with uncontrolled infection. They need to be used carefully in people with liver problems, heart failure, recent heart attack, high blood pressure, diabetes, and in people with psychiatric disturbance (including psychoses and severe affective disorders). We advise our patients of the risks and benefits of corticosteroids to help them decide whether they wish to take them. Special consideration of this balance is needed for pregnant women.

## Conclusion

Over the past decade, the role of ophthalmologists in diagnosing and managing ON has evolved significantly. The diverse causes of ON, many of which require urgent intervention, have highlighted the criticality of timely management in ON, akin to stroke care, under the principle of “time is vision”. Effective collaboration with neuro-ophthalmologists or neurologists is essential for specialised investigation and treatment. Ophthalmologists have a vital role to play in advancing the differential diagnosis, taking the time-critical first decisions about investigation and management and identifying conditions that may arise as complications of treatment.

## Summary

### What was known before:

- The **1992 Optic Neuritis Treatment Trial** provided evidence that corticosteroid therapy has no clear role (based on visual acuity outcome alone) in the management of a first episode of acute optic neuritis (ON), typical of demyelination associated with Multiple Sclerosis. However, it left many questions critical to the acute management of ON more generally, and especially ON not associated with MS, unaddressed.
- For many ophthalmologists, especially those working without rapid access to neuro-ophthalmic support, making a call about whether and when to start high dose corticosteroid therapy, and how to investigate and manage patients with ON, may feel challenging.

### What this article adds:

- In this article, we summarise the latest evidence and provide our neuro-ophthalmic perspective. We share practical and pragmatic tips on how we approach the acute investigation and management of ON in our centres in the UK.
- We highlight our opinion that, ‘*Time is Vision*,’ and share clinical examples which illustrate how safe and rapid control of inflammation with urgent high dose corticosteroid, even inflammation associated with infection, can support preservation of ON axons and good visual outcomes for patients otherwise at risk of rapid and irreversible vision loss.

### Supplementary information

Online Supplement
