# Supplementary material for: How far should I manage acute optic neuritis as an ophthalmologist? A United Kingdom perspective
Source: Eye (Lond). 2024 Jun 12;38(12):2238–45. doi: 10.1038/s41433-024-03164-4 (PMC11306244; doi:10.1038/s41433-024-03164-4)
Supplement: Supplementary file 1 — Online Supplement [file 41433_2024_3164_MOESM1_ESM.pdf]

Online Supplement

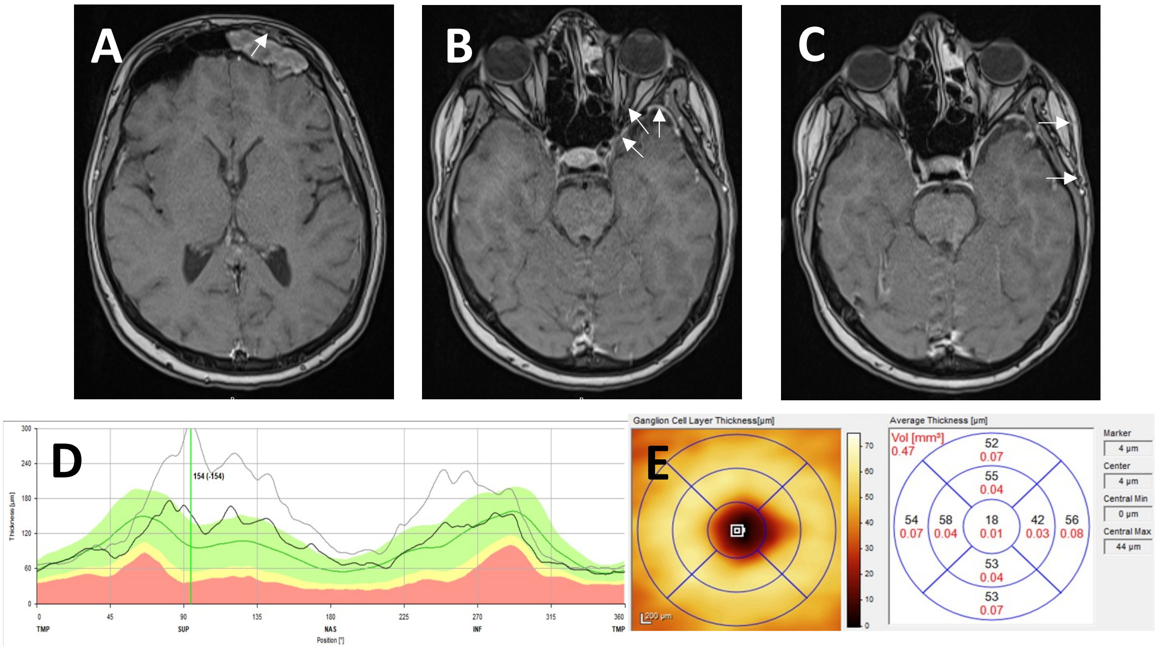

Supplementary Figure 1: Optic neuritis secondary to osteomyelitis associated with severe (presumed) bacterial fronto/maxillary sinusitis: an example of adjunctive corticosteroid therapy with good visual outcome A 23-year-old breast-feeding woman presented with a 2-week history of worsening left frontal/parietal headache, with left facial swelling, blurred left vision and pain on eye movement on the left. On examination, visual acuity was 6/12 left, compared to 6/6 right and she read 9/13 Ishihara plates in both eyes. Slit lamp biomicroscopy and Heidelberg Spectralis optical coherence tomography (OCT) imaging demonstrated unilateral left optic disc swelling with mild posterior vitreous inflammation. She was admitted under Ear Nose and Throat and initiated on IV chloramphenicol 1g 4 times daily for 5 days (metronidazone allergy), with negative septic screen. Her baseline white blood cell count was 19.5 x 10^9/litre (neutrophils 16.6 x 10^9/litre). Nasoendoscopy excluded an invasive fungal infection (e.g. Mucor). Blood cultures and cerebrospinal fluid (CSF) analysis were negative. Baseline Magnetic Resonance Imaging of brain and orbits with contrast demonstrated a subperiosteal abscess overlying the left frontal sinus (Image A), left eye optic neuritis/peri neuritis secondary to parietal dural thickening and enhancement, suggestive of inflammation (Image B) and osteomyelitic changes of the calvarium, left superior frontal and parietal bones (Image C). There was complete opacification of the left maxillary sinus and ethmoidal air cells. She subsequently had extraction of the upper left premolar and maxillary exploration and wash-out. She was effectively treated with combination therapy including IV meropenem 2g tds for 10 days and then IV ertapenem once daily for 4 weeks. Intravenous dexamethasone 6.6mg three times daily was added 2-days after presentation for 2 weeks, followed by oral prednisolone taper from 40mg once daily. At 1-month follow-up, visual acuity and colour vision were improving. The OCT optic disc retinal nerve fibre layer thickening had markedly improved compared to baseline (Image D) while the macular ganglion cell layer was preserved (Image E).

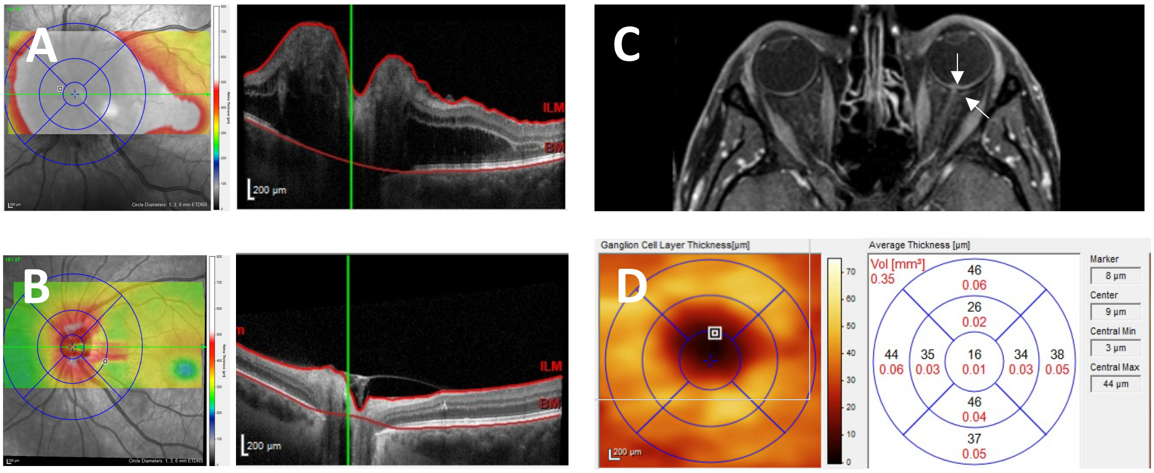

Supplementary Figure 2: Optic neuritis associated with syphilis : an example of good visual outcome following early steroid initiation. A 44-year-old man presented with well-controlled HIV (normal CD4 count, on Highly Active Anti-retroviral Therapy) and a 5-day history of rapid onset painless loss of central vision in the left eye, to 6/24 Snellen acuity. Slit lamp biomicroscopy and Heidelberg Spectralis optical coherence tomography (OCT), with enhanced depth imaging, confirmed severe left optic disc swelling (Image A). We requested blood tests and urgent magnetic resonance imaging (MRI) of brain and orbits with contrast. After 5 days of oral methylprednisolone 500mg once daily, the visual acuity improved to 6/6 with full colour vision on Ishihara plates (13/13). OCT demonstrated a marked improvement in optic disc swelling (Image B). The treponema serology returned positive with a rapid plasma regain (RPR) titre of 1:32 and he commenced 14 days of IM benzylpenicillin 1g. MRI was performed 18 days after presentation and treatment initiation, and showed focal intense enhancement at the left optic nerve, with signal alteration and diffusion abnormality. There was also modest enhancement within the posterior ocular coat, and modest asymmetric reduction in clarity of retrobulbar fat planes. At 3-months follow-up, visual function was stable with visual acuity left eye 6/7.5 and 11/13 Ishihara plates identified, with subjective report of paracentral field distortion and discolouration. OCT demonstrated mild thinning of the retinal ganglion cell layer (Image D).
